# The Innate Immune Playbook for Restricting West Nile Virus Infection 

**DOI:** 10.3390/v5112643

**Published:** 2013-10-30

**Authors:** Kendra M. Quicke, Mehul S. Suthar

**Affiliations:** Department of Pediatrics and Children’s Healthcare of Atlanta and Emory Vaccine Center, Emory University School of Medicine, Atlanta, GA 30329, USA; E-Mail: kquicke@emory.edu

**Keywords:** flavivirus, pathogenesis, encephalitis, RIG-I-like receptor, Toll-like receptor, Nod-like receptor, antiviral gene, innate immunity, interferon, West Nile virus

## Abstract

West Nile virus (WNV) is an emerging mosquito-borne flavivirus that causes annual epidemics of encephalitic disease throughout the world. Despite the ongoing risk to public health, no approved vaccines or therapies exist for use in humans to prevent or combat WNV infection. The innate immune response is critical for controlling WNV replication, limiting virus-induced pathology, and programming protective humoral and cell-mediated immunity to WNV infection. The RIG-I like receptors, Toll-like receptors, and Nod-like receptors detect and respond to WNV by inducing a potent antiviral defense program, characterized by production of type I IFN, IL-1β and expression of antiviral effector genes. Recent research efforts have focused on uncovering the mechanisms of innate immune sensing, antiviral effector genes that inhibit WNV, and countermeasures employed by WNV to antagonize innate immune cellular defenses. In this review, we highlight the major research findings pertaining to innate immune regulation of WNV infection.

## 1. Introduction

### 1.1. West Nile Virus is a Serious Public Health Concern

West Nile virus (WNV) is a member of the Japanese Encephalitis virus (JEV) antigenic complex, which includes JEV, St. Louis Encephalitis, Murray Valley Encephalitis, and other neurotropic mosquito-borne flaviviruses, that combined, are major causes of virus-induced encephalitis throughout the world [1]. Since its introduction into the United States in 1999, WNV has caused over three million infections resulting in over 37,000 confirmed cases and 1,500 deaths [2]. In Europe, annual outbreaks of lineage 1 WNV infections have recently been compounded by the emergence of a pathogenic lineage 2 WNV strain, raising a new threat from a strain that has traditionally been nonpathogenic in humans [3]. Despite the ongoing risk to public health, there are still no approved specific therapeutics or vaccines for use in humans to combat or prevent WNV infection. 

### 1.2. WNV Biology and Pathogenesis

WNV is a member of the *Flaviviridae* family and possesses a positive-sense single-stranded RNA genome of approximately 11 kb in length. The genome consists of three structural proteins (C, prM/M, and E), which mediate virus attachment, entry, and encapsidation and seven non-structural proteins (NS1, NS2A, NS2B, NS3, NS4A, NS4B, and NS5), which participate in viral RNA synthesis. Virus replication occurs in the cytoplasm of infected cells (as reviewed in [4]). Following receptor-mediated endocytosis, genomic viral RNA is immediately translated as a single polyprotein that is co- and post-translationally processed by host and viral proteases to form mature viral proteins. The NS proteins, including the viral RNA-dependent RNA polymerase NS5, form a replication complex that synthesizes negative-sense RNA intermediates, which subsequently serve as the template for synthesis of positive-sense genomic RNA. The new genomes are packaged by the capsid protein (C) into immature virions, which are transported through the cellular secretory pathway, where the host protease furin cleaves prM into its final form (M). These newly generated mature virions then continue to the plasma membrane and are released by exocytosis.

## 2. The Innate Immune Players

The innate immune system encodes a series of pattern recognition receptors (PRRs) that, upon recognition of a viral pathogen-associated molecular pattern (PAMP), induce a potent antiviral host response characterized by production of type I interferon (IFN), IL-1β, and other proinflammatory cytokines, expression of antiviral effector genes, activation of innate immune sentinel cells, and programming protective humoral and cell-mediated immunity [5]. The major PRRs encoded by nearly every mammalian cell are the retinoic-acid inducible gene-I (RIG-I)-like receptors (RLR), Toll-like receptors (TLR), and the nucleotide oligomerization domain (Nod)-like receptors (NLR). These receptors drive complementary antiviral defense programs and each has been implicated in controlling immunity and protection against WNV infection. 

### 2.1. RIG-I-like Receptor Signaling

**Figure 1:**
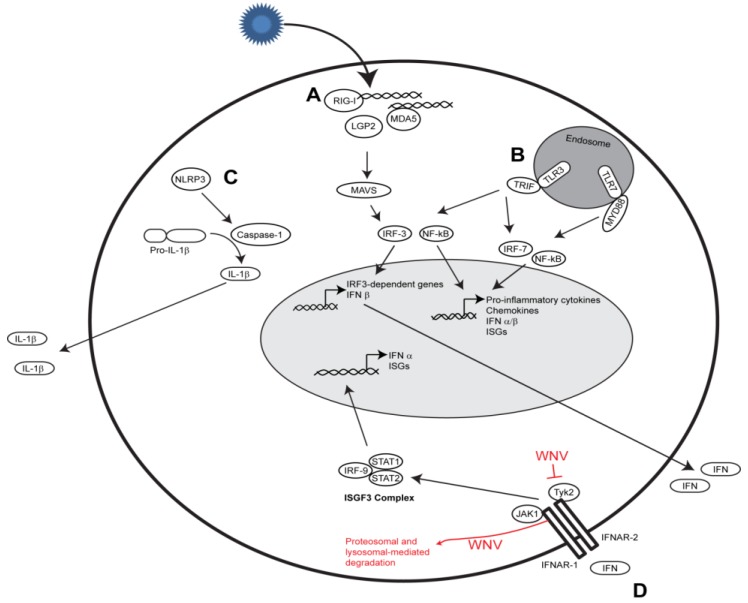
Innate immune signaling. (**A**) Following non-self RNA binding, RIG-I and MDA5 interact with MAVS to induce the expression of IFN-β and IRF-3 target genes; (**B**) TLR3 and TLR7 bind to non-self RNA to trigger IRF-3, IRF-7, and NF-κB dependent gene expression; (**C**) NLPR3 interacts with ASC, leading to caspase-1 activation and IL-1β processing; (**D**) Secreted type I IFN leads to formation of the ISGF3 complex that translocates to the nucleus and induces expression of interferon-stimulated response element (ISRE)-dependent genes, including IRF-7, IFN-α subtypes, and ISGs. Viral proteins that antagonize these pathways are indicated.

The RLRs are a family of cytosolic RNA helicase proteins comprised of three structurally related members: RIG-I, myeloma differentiation antigen 5 (MDA5), and LGP2 [5] (Figure 1). RIG-I and MDA5 consist of two N-terminal tandem caspase-activation and recruitment domains (CARDs), an RNA helicase domain, and a C-terminal repressor domain. In contrast, LGP2 lacks the tandem N-terminal CARDs but does possess an RNA helicase domain and a repressor domain. The CARDs play a pivotal role in signal transduction and, as such, LGP2 has been reported as a regulator, rather than an initiator, of RLR signaling [6,7,8]. Following detection and binding to a non-self RNA ligand in the cytoplasm, RIG-I and MDA5 are post-translationally modified and translocate to mitochondria and mitochondrial-associated membranes [9,10,11,12]. Here, RIG-I and MDA5 interact with the mitochondrial antiviral signaling (MAVS) adaptor protein, which leads to the formation of the MAVS-signalosome, comprised of RLR signaling adaptors, protein kinases, and transcription factors (interferon regulatory factors-1, -3, -5, -7, and NF-κB; [13,14,15,16]). Activated transcription factors translocate to the nucleus and drive transcription of IFN-β, IFN-α4, proinflammatory cytokines, and interferon-stimulated genes (ISGs) that aid in cellular defense against viral infection (as reviewed in [5]). 

The importance of the RLR signaling pathway in protection against WNV has been demonstrated by several studies *in vivo*. The absence of MAVS leads to increased peripheral and central nervous system (CNS) viral burden, enhanced tissue tropism, and enhanced CNS pathology [17]. *RIG-I^−/−^* or *MDA5^−/−^* WNV-infected mice show increased mortality compared to wild type (WT) controls, however, infection of mice lacking both RIG-I and MDA5 results in similar virulence to that of *MAVS^−/−^* mice [18,19]. WNV-infected *LGP2^−/−^* mice also display enhanced susceptibility but this was linked to a defect in T cell responses rather than a major defect in innate immunity [8]. Additional studies have found other RLR signaling factors, such as caspase-12, to be essential for up-regulating defenses against WNV [20]. Mice lacking caspase-12 (*Casp12^−/−^*) experienced higher mortality and more severe neurological effects than WT mice. Correspondingly, *Casp12^−/−^* mice had greater viral load in the CNS and higher viremia than WT mice. Subsequent studies *in vitro* found that caspase-12 regulates type I IFN production by interacting with RIG-I and regulating TRIM25-mediated ubiquitination in WNV-infected cells.

RLR signaling protects against WNV infection by triggering a potent innate immune response. In the absence of MAVS, the key *in vivo* target cells of WNV infection—macrophages, dendritic cells, and cortical neurons—displayed abrogated type I IFN and related antiviral responses that correspond to uncontrolled virus replication [17]. The contributions of the individual RLRs have recently been examined and RIG-I was determined to be important for triggering the early innate immune response, whereas MDA5 functions during the later stages of infection to enhance innate immune signaling [21,22]. LGP2, in contrast, plays a subtle role in promoting RLR signaling in response to WNV [8]. While the specific WNV PAMP signature that activates RIG-I or MDA5 is not known, Shipley and colleagues determined that multiple RNA segments within both the WNV genome and antigenome encode non-self moieties that can trigger RIG-I signaling [23]. In support, a study by Errett and colleagues found that RNA isolated from WNV-infected cells at early time points post-infection activated RIG-I signaling whereas RNA isolated at later times induced MDA5 signaling [18]. Additionally, the RLR stimulatory RNA from infected cells was determined to be double-stranded and contain a triphosphate moiety. Further investigation is warranted to define a specific PAMP signature that leads to activation of RIG-I or MDA5 signaling. However, WNV passively evades RLR detection at early times during infection [21], though the underlying mechanisms are not well understood. A recent study has suggested that viral replication complexes are strategically sequestered within vesicles derived from the endoplasmic reticulum, also called vesicle pockets (VP) [24,25]. The interiors of these VPs are thought to contain products of viral replication, including nonstructural viral proteins, *de novo* synthesized RNA and double-stranded RNA intermediates. A similar mechanism has also been suggested for the closely related mosquito-borne flavivirus dengue virus (DENV; [26]).

### 2.2. Toll-like Receptor Signaling

The TLRs 3, 7, and 8, unlike the RLRs, reside in endosomal vesicles and recognize distinct viral and bacterial PAMPs. TLR3 recognizes dsRNA, whereas TLR7/8 recognize GU-rich ssRNAs [27,28,29]. Upon binding PAMP RNA, TLR3, and TLR7/8 recruit adaptor proteins and activate latent transcription factors that lead to the production of type I IFN and proinflammatory cytokines [30,31]. 

Both TLR3 and TLR7 are important in regulating immunity to WNV but, unlike the RLRs, function in a cell- and tissue-specific manner. TLR3 signaling in cortical neurons, but not macrophages or DCs, promotes type I IFN production and is required for controlling virus replication [32]. As expected, within the CNS, TLR3 was also found to be important for controlling virus replication, however it is still not entirely clear how TLR3 signaling influences CNS immunity when challenged with WNV. One possibility is that TLR3 signaling transiently alters blood-brain-barrier permeability [33], although this finding still remains controversial. TLR7 signaling is important for triggering type I IFN and proinflammatory cytokine production within neurons, macrophages, and keratinocytes, but not DCs [34,35]. *In vivo*, TLR7 and Myd88 signaling are important for regulating immune cell migration [34,36]. Within keratinocytes, TLR7 signaling regulates proinflammatory cytokine production, including IL-6, IL12, and IL1β, and directs Langerhans cell migration from the skin to the draining lymph node [35]. Similarly, triggers from TLR7 and Myd88 control CNS-intrinsic IL-12 and IL-23 signaling, which initiates responses that are vital for macrophage and leukocyte homing to the CNS [36]. More recently, immunization studies with a candidate WNV vaccine, comprised of a virus-like particle that can only undergo one round of replication, revealed that TLR3 and Myd88-dependent signaling is important for promoting development of germinal centers, long-lived plasma cells, and memory B cells [37]. Combined, these studies show that TLR3 and TLR7 function in a cell- and tissue-specific manner to regulate immunity to WNV. 

Presently, it is inconclusive whether WNV has evolved efficient strategies to evade TLR3 or TLR7 signaling. The WNV NS1 protein, which is localized to the lumen of the endoplasmic reticulum and secreted from infected cells, was shown to antagonize TLR3 signaling [38]. In this study, over-expression of WNV NS1 was found to disrupt TLR3-mediated activation of IRF-3, NF-κB, and production of proinflammatory cytokines. However, an equally compelling study found that WNV NS1, in addition to the NS1 proteins from DENV and the related Yellow Fever virus, failed to disrupt TLR3 signaling [39]. In this study, Baronti and colleagues did not observe protein-protein interaction or colocalization between WNV NS1 and TLR3 or make the observation that over-expression of WNV NS1 inhibits IFN-β transcription. Thus, the finding that WNV NS1 antagonizes TLR3 signaling remains controversial. 

### 2.3. Nod-like Receptor Signaling

The inflammasome is an innate immune signaling complex comprised of cytosolic PRRs (34 NLR genes in mice and 22 NLR genes in humans) that detect ‘danger signals’ within the cellular environment, including microbial PAMPs and cell stress products (as reviewed in [40]). Inflammasome activation is regulated by two signals: ‘signal 1’—a priming signal to induce pro-IL-1β and pro-IL-18 expression which can be mediated by the TLR, RLR, or NLR signaling pathways; and ‘signal 2’—a maturation signal wherein the inflammasome complex, comprised of an activated NLR, apoptosis-associated speck-like protein containing a CARD (ASC) adaptor protein and caspase-1, processes pro-IL-1β and pro-IL-18 into their mature forms which are subsequently secreted from the cell. These cytokines promote immune cell activation and trafficking to sites of infection, and also drive a programmed cell death response known as pyroptosis.

Cell culture-based studies have revealed that WNV activates the NLRP3 inflammasome complex and induces IL-1β secretion [41]. In support, symptomatic human cases of WNV display enhanced systemic IL-1β over the course of WNV disease, suggesting that IL-1β may play an important role in mediating protective immunity. Indeed, the IL-1 receptor, NLRP3, ASC, and caspase-1 are important in mediating protection against WNV infection [41,42,43]. Within the CNS, IL-1β signaling promotes DC activation, CD8^+^ T cell reactivation and effector responses, regulates antiviral signaling within neurons, and limits neuronal cell death. Other investigations indicated that IL-1β signaling is important for migration of Langerhans cells from the epidermis to the draining lymph node following cutaneous WNV infection [35,44]. Collectively, these findings indicate that IL-1β must achieve a fine balance between immune-mediated protection and immune-mediated pathology during WNV infection. Several questions still remain unanswered, including the viral PAMP or cellular stress that activates inflammasome signaling, molecular mechanisms of IL-1β signaling within the CNS, and antiviral signaling induced by IL-1β. 

## 3. IFN Antiviral Responses and Viral Countermeasures

Type I interferon (IFN) signaling triggers a potent antiviral response culminating in the rapid expression of a multitude of ISGs that serve to restrict virus replication and spread within the host (Figure 1). IFN-α and IFN-β are secreted from the cell and bind to the IFN-α/β receptor (IFNAR) complex in an autocrine or paracrine manner. This leads to phosphorylation of tyrosine kinase 2 (Tyk2) and Janus kinase 1 (Jak1), activation of signal transducer and activator of transcription 1 (STAT1), STAT2, and IRF-9, and formation of the interferon-stimulated gene factor 3 (ISGF3) complex. This complex translocates to the nucleus and binds specific DNA sequences known as interferon stimulated response elements (ISRE) to initiate production of antiviral ISGs [45]. 

Type I IFN is critical for controlling WNV replication and mediating protection against infection. Original studies from the 1950s demonstrated that type I IFN has potent antiviral activity against WNV infection [46]. Subsequent studies have shown that type I IFN signaling is necessary for protection, controlling virus replication, and restricting tissue tropism [47,48]. Furthermore, mice that are unable to produce IFN-β show increased susceptibility accompanied by uncontrolled virus replication within the periphery and CNS [49]. In addition to its role in innate immunity, type I IFN signaling contributes to early B cell activation [50], shaping anti-WNV CD8^+^ T cell effector responses [51], and controlling regulatory T cell responses [49]. While type I IFNs seem to be the stronger inducers of antiviral activity, one study has also implicated IFN-λ, a type III IFN, in anti-WNV activity *in vitro*. IFN-λ seems to play a modest role in preventing infection of cells by WNV virus-like particles (VLPs), but does not appear to be effective at inhibiting virus replication [52].

As a countermeasure, WNV utilizes strategies to antagonize type I and type III IFN signaling. Several mechanisms to block type I IFN responses have been presented, including: (1) inhibition of Tyk2 phosphorylation [53]; (2) lysosomal- and proteosomal-mediated degradation of IFNAR1 [54]; (3) redistribution of cellular cholesterol [55]; and (4) activation of the unfolded protein response [56,57]. Both the structural and nonstructural genes have been implicated with antagonizing type I IFN signaling [58], though for many of these, investigation is still underway to determine the precise mechanism of action. NS2A, NS2B, NS3, NS4A, and NS4B appear to inhibit phosphorylation of STAT1 and STAT2, preventing their translocation to the nucleus and the subsequent production of ISGs [59,60,61]. NS5 has also been implicated in inhibition of STAT1 phosphorylation, but may additionally be involved in the degradation of STAT2 [62]. More recently, it was demonstrated that synthesis of the viral non-coding subgenomic RNA (sfRNA) interferes with type I IFN signaling [63], although the mechanism of action is not well understood. WNV appears to efficiently block IFN-λ signaling by preventing IFN-λ-induced phosphorylation of Tyk2, STAT1, and STAT2 [52]. It has been suggested that this inhibition may occur at the IFN-λ receptors, but further studies are needed to be conducted to test the validity of this hypothesis.

### 3.1. Cell- and Tissue-Specific Regulation

Recent research efforts by several groups have focused on understanding how type I IFN signaling and subsequent ISG expression is regulated in a context-dependent manner. Our group recently found that ISGs are differentially expressed within the spleen (permissive) and the liver (nonpermissive) during WNV infection [48]. Similarly, two distinct neuronal subsets within the CNS show differences in sensitivity to type I IFN and permissiveness to WNV infection [64]. Through a microarray analysis, granule cell neurons of the cerebellum were found to differentially express Ifi27, Irg1, and Rsad2. Over-expression of these ISGs in cortical neurons reduced WNV replication. Differential gene expression between these two neuronal subsets was linked to epigenetic modification and regulation by microRNAs. Combined, these results reveal the complexities of innate immune signaling during virus infection and underscore the importance of understanding the cell- and tissue-specific regulation of innate immune responses. 

### 3.2. ISG Screens

Several groups have recently reported results from ISG screening assays, which identified genes with antiviral activity against WNV. Gain-of-function based approaches, which utilize lentiviruses or plasmids to ectopically express ISGs, identified several novel antiviral effector genes that inhibit WNV infection. A small-scale screen by Jiang and colleagues identified RSAD2 (also known as viperin) and ISG20 as cellular enzymes that efficiently suppress WNV infection [65]. In this analysis, over-expression of viperin and ISG20 suppressed WNV-replicon colony formation, suggesting that these antiviral proteins likely target viral RNA or protein biosynthesis. Using a similar over-expression based approach, Schoggins and colleagues screened more than 380 human genes and identified several additional ISGs, including PRRs (RIG-I, MDA5, CGAS), transcription factors (IRF1, ATF3, IRF7), and uncharacterized antiviral genes (HPSE, NAMPT, PBEF1, SAA1, and PHF15) that suppressed WNV replication [66]. Loss-of-function based approaches, which involve gene silencing through the use of short-hairpin RNA (shRNA) or small-interfering RNA (siRNA), have also been employed to identify anti-WNV ISGs. Using this approach, Krishnan and colleagues identified 22 novel genes that, when silenced, enhanced WNV replication [67]. Many of these genes are uncharacterized and it has yet to be determined how they restrict virus replication. More recently, Li and colleagues used shRNAs to screen 245 human ISGs and identified 47 that demonstrated antiviral effects against WNV infection [68]. This list of ISGs includes previously identified genes (e.g., MAVS, STAT2, IRF1, IFITM2, and PKR) as well as novel ISGs such as DDX24, IFI44L, IFI6, TRIM21, and TRIM6. Other approaches have used a combination of global transcriptional profiling and biological validation to identify and characterize candidate antiviral effector genes. In one such study, Qian and colleagues identified AIM2, IFI27, CCR3, and CXCR3 as WNV restriction factors [69]. Combined, these studies have identified several antiviral effector genes that inhibit WNV infection, but much still remains to be understood about how they mechanistically suppress viral infection. Further studies should place a greater emphasis on using high-throughput technologies, biological validation, and mechanistic analysis to better define the mode of action for how ISGs suppress WNV infection. 

### 3.3. ISGs that Inhibit WNV Infection

Protein kinase R (PKR) is a cytosolic serine/threonine kinase that recognizes viral double-stranded RNA. Activated PKR phosphorylates eIF2α on Ser51, which attenuates cap-dependent translation and leads to stress granule formation [70]. Mice that lack both PKR and the endoribonuclease of the 2',5'-oligoadenylate synthase pathway (RNaseL) show reduced survival accompanied by altered tissue tropism, and reduced viral control within the spleen and CNS as compared to RNaseL-deficient mice [71,72]. While this does not directly demonstrate an antiviral function for PKR, subsequent studies have shown that PKR is important for regulating type I IFN induction in response to WNV infection [73]. The WNV subgenomic RNA and stem-loop structures within the 5' and 3' untranslated regions can activate PKR and lead to eIF2α phosphorylation [74]. However, it has also been suggested that WNV evades PKR activation and stress granule formation by suppressing early viral RNA synthesis [74]. Intriguingly, WNV uses a similar strategy to evade early detection by the RLRs, suggesting a common mechanism is potentially employed to evade cytosolic PRRs detection. 

The IFN-inducible tetratricopeptide family of genes, comprised of IFIT1, IFIT2, IFIT3, and IFIT5, inhibit virus infection through multiple mechanisms, including translation inhibition through binding translation factors, binding and sequestering viral RNAs containing a 5' triphosphate, directly interacting with viral proteins and disrupting an essential viral process, or promoting cellular apoptosis (as reviewed in [75]). The IFIT genes are robustly induced following WNV infection [76,77,78]. IFIT1, which has potent antiviral activity against a number of viruses, does not inhibit WNV [77]. Interestingly, a mutant virus that lacks 2'-O methlytransferase activity, was highly sensitive to IFIT1 [79], demonstrating that WNV employs a unique strategy to evade the antiviral effects of IFIT1. In contrast, IFIT2 possess antiviral activity against WNV, as cells that lack IFIT2 show increased virus replication [76]. Although required for protection against WNV, IFIT2 is important for controlling virus replication within the CNS, but not the periphery, suggesting a cell- and tissue-specific regulatory mechanism for controlling virus replication [80]. IFIT3 also has been shown to inhibit WNV infection [66], but neither the mechanism of action nor its role in viral pathogenesis are well understood. 

The IFN-induced transmembrane (IFITMs) family of genes, not to be confused with the IFIT genes, consists of three small transmembrane proteins (IFITM1, IFITM2, and IFITM3) that are important in governing cellular processes (as reviewed in [81]). The IFITM proteins also inhibit a number of viruses, including WNV. Brass and colleagues found that over-expression or silencing expression for all three IFITM genes alters WNV replication [82]. In subsequent experiments, IFITM2 and IFITM3, but not IFITM1, were able to inhibit WNV infection. It is still not clear as to the discrepancy between the antiviral activity of IFITM1 between these studies [65,66]. Mechanistically, IFITM2 and IFITM3 did not appear to inhibit WNV-replicon colony formation [65], suggesting that these proteins function at the early stages of the virus life cycle, likely by disrupting virus binding, entry, or uncoating. In support, IFITM1 was recently shown to inhibit viral entry of the related flavivirus Hepatitis C virus [83], suggesting a common mechanism of action for the IFITM genes amongst flaviviruses. 

Viperin is a radical S-adenosylmethionine (SAM) enzyme that can be induced through both IFN-independent and -dependent manners [84]. Ectopic expression of WT viperin, but not an enzymatically inactive mutant, strongly inhibits WNV-replicon colony formation [65], suggesting that viperin and its associated enzymatic activity are important for inhibiting either viral RNA or protein biosynthesis. Similar to IFIT1, viperin is important for protection against WNV infection and controlling virus replication within the CNS [85]. While the antiviral mechanisms are not well understood, it is possible that viperin promotes TLR7 signaling [86], affecting the formation of replication complexes [87], or altering WNV protein translation through its association\localization with the endoplasmic reticulum [88]. These possibilities as well as others should be examined to better define the precise mechanism of action used by viperin to restrict WNV replication. 

The 2'-5'-oligoadenylate synthase (OAS)/RNaseL proteins are a class of IFN-inducible PRRs that can recognize dsRNA and restrict a number of viruses, including WNV [89]. Upon binding dsRNA, OAS produces 2'-5'-linkled oligoadenylates that activate latent RNaseL to degrade viral and cellular ssRNA [90]. These RNaseL cleavage products can also serve as RLR ligands to amplify innate immune signaling and antiviral defenses [91,92]. The RNaseL pathway is important for controlling virus replication and mediating protection against WNV infection [63,71,72]. Importantly, the *Oas1b* allele, which encodes an inactive 2'-5' OAS, has been classified as a major flavivirus resistance gene in mice [72,89,93,94]. Similarly, the *OAS1* gene has been identified in humans and horses as a risk factor for symptomatic WNV infection [95,96]. Ectopically expressed Oas1b increases resistance to WNV [97]. Although the exact mechanism of this restriction is not defined, Oas1b may act as a negative regulator of Oas1a activity and reduce synthesis of 2'-5'oligoadneylates [94]. In addition, Oas1b localizes to the endoplasmic reticulum during WNV infection and is part of a complex of proteins comprised of oxysterol binding protein-related protein 1L (ORP1L), which is an important host factor for mediating ER-late endosome membrane contacts and late endosome motility, and ATP binding cassette protein 3, subfamily F (ABCF3), a protein with little known function [98]. Thus, Oas1b may function as a negative regulator of RNaseL activity and as a direct antiviral effector for restricting WNV infection.

## 4. Conclusions

WNV continues to pose a significant public health risk throughout the world. Over the past decade, significant advancements have expanded our understanding on the virus-host interactions that govern immunity to infection. These major achievements include identifying the host innate immune sensors, understanding the role of type I IFN and IL-1β, identifying antiviral effector genes, and uncovering countermeasures employed by the virus to antagonize innate immune responses. While these findings certainly hold promise for developing novel treatments or improving immunogenicity during vaccination, several questions still remain to be answered. First, what are the specific viral RNA signatures that trigger RLR, TLR, and NLR signaling? Second, how are the innate immune sensing pathways regulated in a cell- and tissue-specific manner? And third, how exactly does WNV evade PRR detection and block type I IFN signaling? Answers to these fundamental questions and others posed throughout this review will provide valuable insight into the underlying mechanisms of innate immunity and have broad spanning implications across flaviviruses and other RNA viruses. 
